# Supplementary material for: The distinct role of human PIT in attention control
Source: eLife. 2026 Mar 16;14:RP107111. doi: 10.7554/eLife.107111 (PMC12991642; doi:10.7554/eLife.107111)
Supplement: Supplementary file 1. [file elife-107111-supp1.docx]

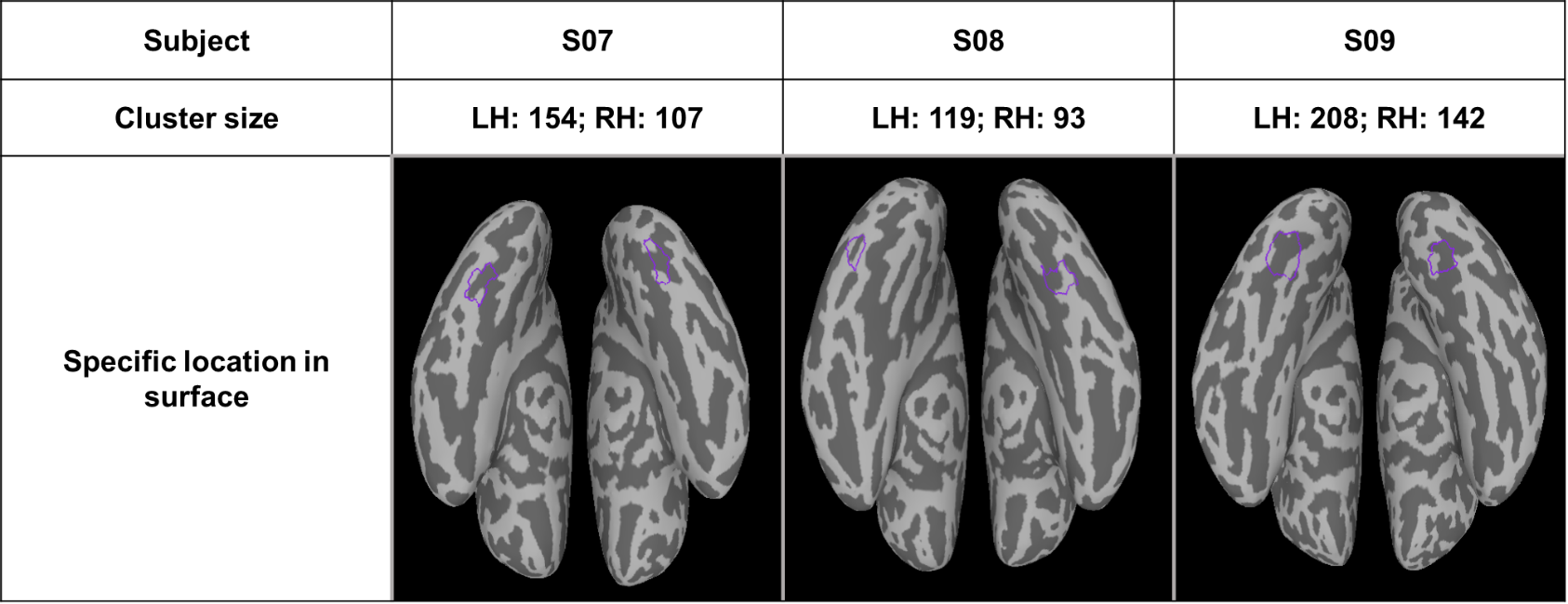

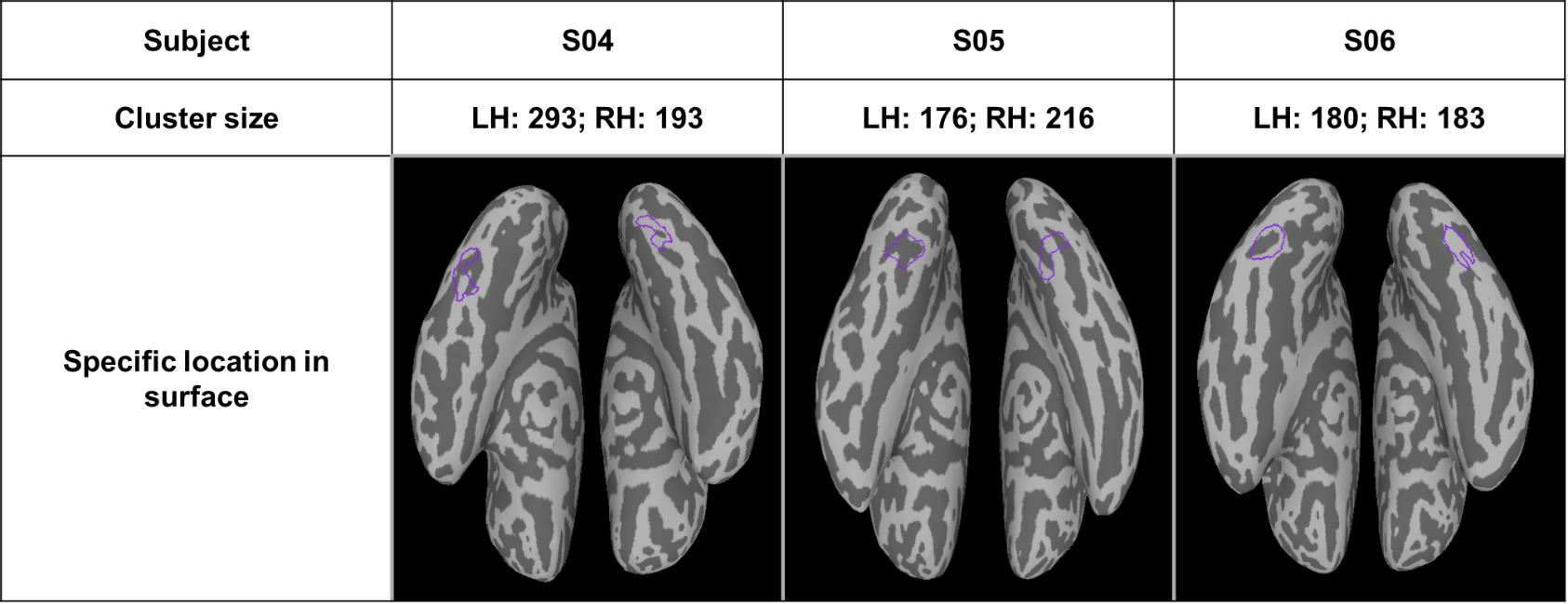

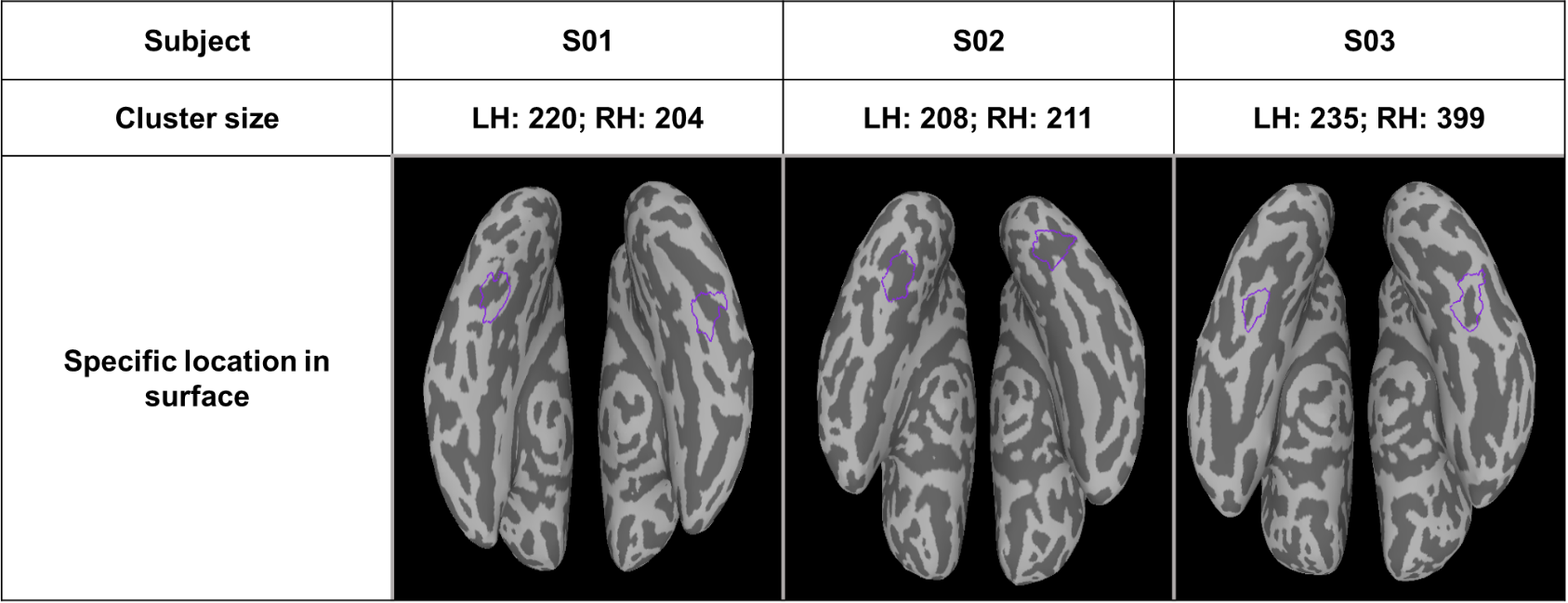


**Supplementary File 1**
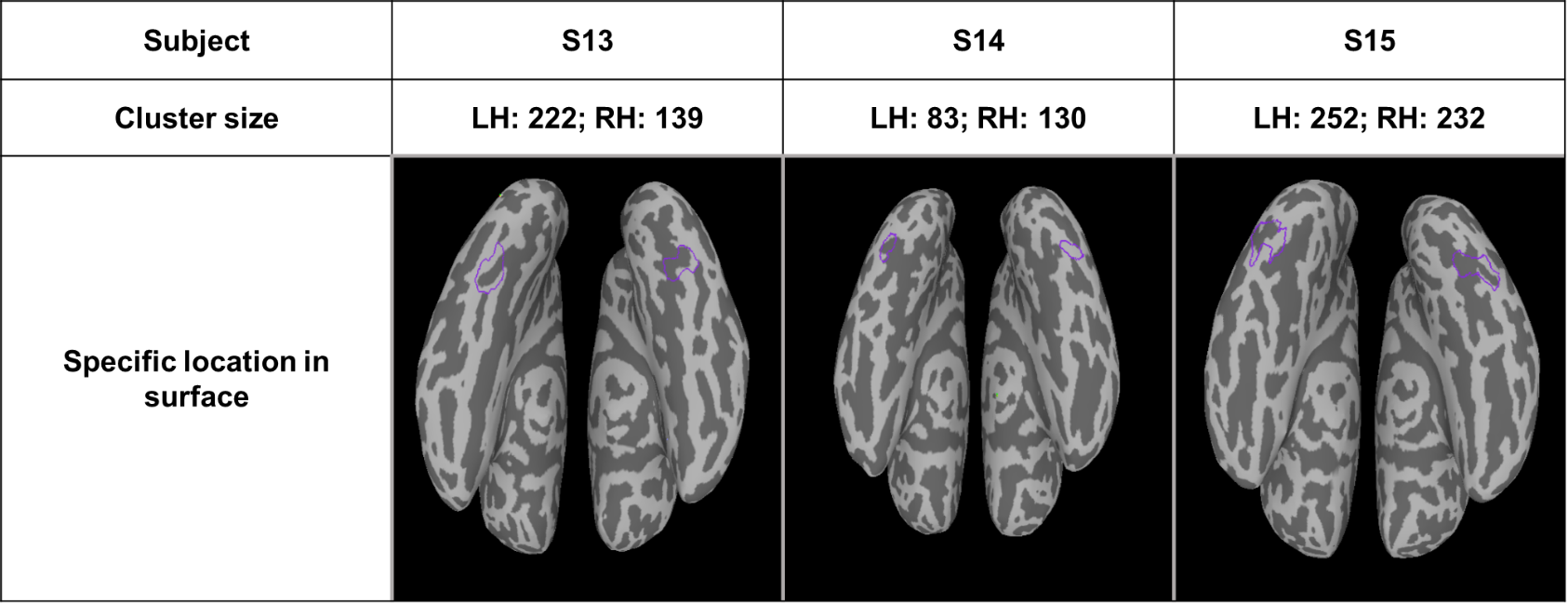

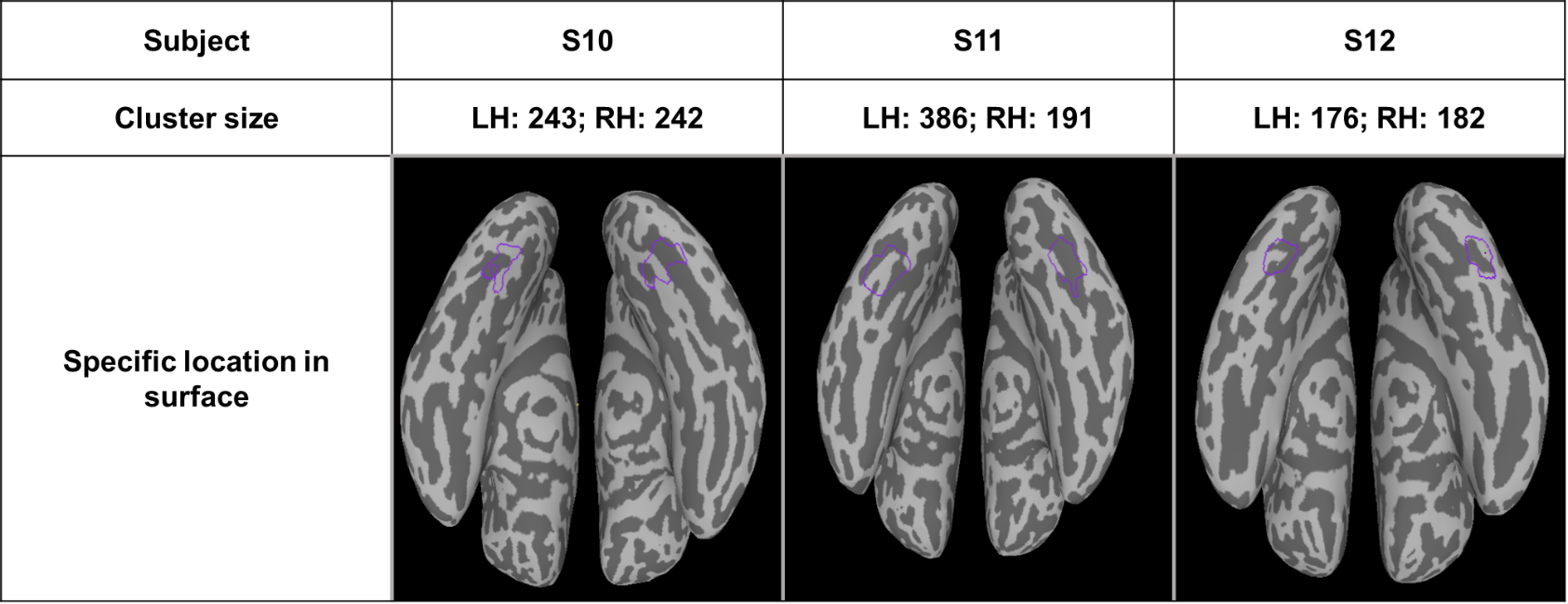
: Exact location and cluster size of hPIT in every subject’s cortical surface. ROI hPIT is selected by purple coil and its cluster size is calculated after projected to volume (one voxel: 2 x 2 x 2 mm).
