# Supplementary material for: The distinct role of human PIT in attention control
Source: eLife. 2026 Mar 16;14:RP107111. doi: 10.7554/eLife.107111 (PMC12991642; doi:10.7554/eLife.107111)
Supplement: Supplementary file 2. [file elife-107111-supp2.docx]

| **Condition**  **ROI** | **Blank** | **Dot** |
| --- | --- | --- |
| **hPIT**  **V1**  **MT**  **IPS_P**  **IPS_A**  **FEF**  **TPJ**  **VFC**  **FFA**  **LOp** | 16.59  7.18  9.46  7.57  2.79  3.96  1.89  4.83  3.06  6.75 | 28.44  8.87  16.94  6.73  2.96  3.08  4.10  4.15  19.98  13.36 |

**Supplementary File 2**: Summary table of attentional modulation (signal change%) across ROIs (hPIT, V1, MT, IPS, FEF, TPJ, VFC, FFA, LOp) under both blank and dot conditions.
