# Supplementary material for: The distinct role of human PIT in attention control
Source: eLife. 2026 Mar 16;14:RP107111. doi: 10.7554/eLife.107111 (PMC12991642; doi:10.7554/eLife.107111)
Supplement: Supplementary file 3. [file elife-107111-supp3.docx]

| **Condition**  **ROI** | **Blank**  **(df=14)** | **Dot**  **(df=14)** |
| --- | --- | --- |
| **V1** | t=3.390, p=0.0044 | t=4.743, p=0.0003 |
| **hPIT** | t=4.747, p=0.0003 | t=8.545, p<0.0001 |
| **MT** | t=3.474, p=0.0037 | t=6.162, p<0.0001 |
| **IPS_P**  **IPS_A**  **FEF**  **TPJ**  **VFC** | t=5.975, p<0.0001  t=2.606, p=0.0207  t=5.504, p<0.0001  t=1.756, p=0.1009  t=4.746, p=0.0003 | t=6.035, p<0.0001  t=5.425, p<0.0001  t=5.202, p=0.0001  t=3.196, p=0.0065  t=6.436, p<0.0001 |

**Supplementary File 3**: Results of one sample t test measuring the modulation of attention by beta value of contrast [attend contralateral – attend ipsilateral].
